# Identification of a potential source of error for 6FFF beams delivered on an Agility^TM^ multileaf collimator

**DOI:** 10.1002/acm2.13212

**Published:** 2021-03-06

**Authors:** Friedlieb H. Lorenz, Matthew I. Paris

**Affiliations:** ^1^ Department of Radiation Oncology Southern District Health Board Dunedin New Zealand

**Keywords:** FFF, MLC, small fields, sweeping gap, VMAT

## Abstract

**Purpose:**

The performance of the Agility^TM^ multileaf collimator was investigated with a focus on dynamic, small fields for flattening filter free (FFF) beams.

**Methods:**

In this study we have developed a simple tool to test the robustness of the control mechanisms during dynamic beam delivery for Elekta’s VersaHD linear accelerator with Integrity 4.0.4 control software. We have programed the planning system to calculate dose for delivery of sweeping gaps. These sweeping gaps have a constant speed, constant size, and are delivered at a constant dose rate. Therefore they specifically identify delivery problems in dynamic mode.

**Results:**

The Elekta Agility^TM^ control mechanism fails to maintain accurate delivery for small, dynamic sweeping gaps. For small gap sizes, the Agility^TM^ control mechanism delivers a field that is more than four times the size of the planned field width without generating an interlock. This has dosimetric implications: The discrepancy between calculated and measured doses increases with decreasing gap size and exceeds 10% and 60% at isocenter for a 3.5 mm and 1 mm gap size, respectively.

**Conclusion:**

A deficiency of the Agility^TM^ control system was identified in this study. This deficiency is a potential source of error for volumetric modulated arc therapy fields and could therefore contribute to relatively high failure rates in quality assurance measurements, especially for FFF beams.

## 1 INTRODUCTION

Modern day radiotherapy frequently utilizes modulated treatment, either in form of static gantry dynamic multileaf collimator (MLC) treatments or volumetric modulated treatment (VMAT),^1^ for which the gantry, MLC leaves, and dose rate constantly change during beam delivery.^2^ Such advanced treatment techniques require a well‐controlled beam delivery during all stages of treatment, a stable beam, and a robust MLC model implemented in the treatment planning system (TPS), especially when consideration is given to reduction of standard planning target volume (PTV) margins in stereotactic body radiotherapy (SBRT).^3^

The demands on the delivery components and TPS model have further been intensified with the arrival of high dose rate delivery, such as flattening filter free (FFF) beams, also because FFF beams with their characteristically peaked profile are generally used for relatively small fields.^4^, ^5^, ^6^, ^7^

Highly modulated treatment deliveries, especially when using small fields and high intensity beams, require robust control mechanisms during beam delivery and advanced beam models in the TPS as the high dose rate accentuates the impact of small delivery errors.

In this study we focus on delivery anomalies of the Agility^TM^ MLC, which could contribute significantly to discrepancies between calculated and measured doses for regular clinical plans.

In order to identify relatively high failure rates on routine quality assurance (QA) measurements, specific MLC test patterns were developed in order to isolate potential root causes. The evaluation of delivered MLC fields has triggered the investigation presented in this manuscript, which focuses on the delivery accuracy of the Agility^TM^ MLC for small aperture in dynamic mode. The same dynamic MLC sequences were also delivered on Varian’s TrueBeam^TM^ for comparison.

## 2 MATERIALS AND METHODS

The 6FFF beam modality on Elekta’s VersaHD^TM^ linear accelerator (linac) with Integrity 4.0.4 control software, was fully commissioned and the corresponding beam model validated in Elekta’s Monaco® TPS, version 5.11.02.^8^ Beam tuning, MLC calibration, and MLC beam modeling in Monaco® were performed according to Elekta’s recommended procedure, taking into account most recent findings.^9^ Performance of these procedures was well within required specifications. MLC parameters in Monaco® were fine‐tuned to achieve optimal agreement for point dose measurement and dose distribution for a range of VMAT and dynamic conformal arc therapy (DCAT) plans.^10^ However, clinical VMAT plans still showed unacceptably low pass rates on the VersaHD^TM^ linac, especially for highly modulated fields and small targets. SunNuclear’s ArcCheck^TM^ was used for these measurements.

### 2.1 Sweeping gap test fields

We have developed test fields, which consist of dynamic MLC (dMLC) sequences. Each sequence represents a gap of constant size, sweeping across the field at constant speed and dose rate. The gap size is different for each field (20 mm, 10 mm, 5 mm, 3.5 mm, 2 mm, and 1 mm). Similar tests have been used,^11^ including their application for FFF beams,^12^ but, to the best of the author’s knowledge, have never been measured on a VersaHD^TM^ with Agility^TM^ MLC.

These fields specifically identify delivery problems in dynamic mode and potential weaknesses of certain parameters in the TPS, such as the leaf offset, leaf tip transmission, interleaf transmission, and MLC scatter.^13^

All fields were delivered with 3000MU, except for the 20 mm gap (2000MU). The dose calculations were performed with Monaco’s XVMC Monte Carlo algorithm using a 1.5 mm grid size and a variance of 0.5% per control point.

These test fields were generated in Monaco 5.11.02 and calculated on a PTW RW3 phantom. All fields were delivered in clinical mode with a nominal dose rate of 1400 MU/min using the Mosaiq® Radiation Oncology patient management system. Measurements were performed using IBA’s MatriXX® Evolution detector and compared with calculated dose distributions.

The service graphing tool was used to evaluate the so‐called “desired” and “actual” leaf positions during beam delivery. The data points were acquired with a sampling rate of 4 Hz.

### 2.2 Clinical plans

Further to these described test fields a range of VMAT and DCAT plans were created by optimizing patient plans in Monaco® using its built‐in sequencer. These plans created by Monaco® were analyzed with in‐house software in order to quantify the contribution of small leaf apertures (≤3.5 mm). All leaf apertures not covered by the diaphragms were quantified for all exposed leaf pairs and segments and weighed with the corresponding MU per segment, following an approach described by Feng *et al* recently.^7^

The MU‐weighed ratio of small apertures (≤3.5 mm) to the total number of apertures was then determined for each plan.

This selection of DCAT and VMAT plans was also measured, using the locally established QA procedure: Measured with SunNuclear’s ArcCheck^TM^ in absolute mode and evaluating the 2%/2 mm as well as the 3%/3 mm gamma criteria. The option to “Apply Measurement Uncertainty” in the corresponding software was selected.

## 3 RESULTS

### 3.1 Sweeping gap test fields

**Fig. 1:**
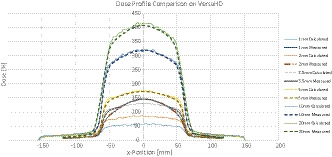
Dose profiles of measured and calculated doses for different gap sizes on the VersaHD.

**Fig. 2:**
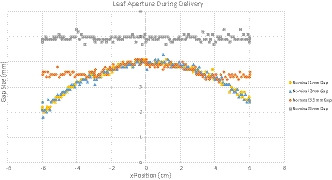
Leaf aperture during delivery extracted from a log file for one representative leaf pair.

It was found that the dose calculation for gap sizes of 20 mm, 10 mm, and 5 mm is in agreement with measurements. The relative dose difference at central axis was −2.1%, −1.0%, and −0.8% for the 20 mm, 10 mm, and 5 mm gap, respectively (negative value indicates lower measured dose). However, for gap sizes 3.5 mm and below significant errors were identified, as shown in Fig. 1: the dose difference at central axis is 10.2%, 41.5%, and 61.3% for the 3.5 mm, 2mm, and 1mm gap, respectively. It is also interesting to observe that measurements for the 1mm and 2mm gaps produced identical results, with the 1mm gap therefore deviating more from calculation. This result indicates a delivery problem for very small gap sizes, which can indeed be confirmed by further analysis of individual leaf positions during delivery, as shown in Fig. 2.

The leaf aperture during delivery confirms inaccurate positioning of leaves for small gap sizes (3.5 mm and smaller). While the DICOM plan requested a 1 mm gap size, the average gap size was 3.4 mm during delivery (minimum = 2 mm, maximum = 4.1 mm). For the 2 mm nominal gap, the delivery was nearly identical with the 1mm nominal gap, also showing a 3.4 mm average gap size (minimum = 1.8 mm, maximum = 4.3 mm). The 3.5 mm gap was closer to its nominal size with an average gap size of 3.6 mm (minimum = 3.2 mm, maximum = 4.1 mm).

No delivery interlock was generated by the linac. Tests were also performed at a lower dose rate to determine whether leaf speed contributed to the failure of the MLC control system to generate a positional interlock. But even at a dose rate of 150 MU/min no interlock was generated.

The gap size itself does not reveal the source of this error. Comparison of planned leaf positions and actual leaf positions will determine which leaf bank introduces the error.

**Fig. 3:**
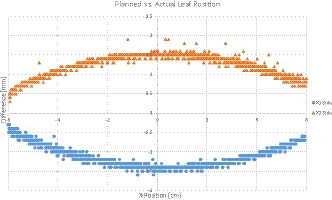
Planned versus actual leaf position during the delivery of the 1mm gap for a representative leaf pair.

Figure 3 shows the planned vs. actual leaf position during the delivery of the 1mm gap for a representative leaf pair. It can be clearly seen that neither leaf bank reaches their planned position at any time during treatment. X1 is constantly too far ahead of its planned position and X2 is constantly lagging behind its planned position. The difference increases as the aperture approaches the central axis and reaches −1.5 mm (maximum −1.6 mm) and +1.5 mm (maximum 1.9 mm) for X1 and X2, respectively. This introduces a total error of 3mm for the 1mm gap size, therefore quadrupling the field width.

**Fig. 4:**
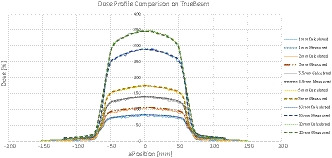
Dose profiles of measured and calculated doses for different gap sizes on the TrueBeam^TM^.

In comparison, the TrueBeam^TM^ delivered all these fields according to plan and dosimetric agreement was well within specifications for all gap sizes (see Fig. 4).

The relative dose difference at central axis was 0.03%, 0.14%, −1.19%, 2.2%, 1.32%, and 3.0% for the 20 mm, 10 mm, 5 mm, 3.5 mm, 2 mm, and 1 mm gap sizes, respectively.

### 3.2 Clinical plans

As the Agility^TM^ seemed unable to maintain accurate leaf positioning for aperture widths of less than 3.5 mm, other Monaco® plans were analyzed in terms of aperture widths and passing rates.

**Fig. 5:**
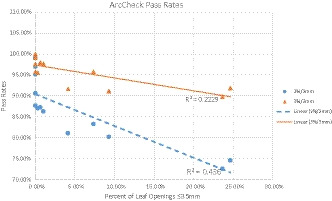
Measurement results in relation to small apertures.

The measurement results in terms of their ArcCheck^TM^ passing rates of a selection of DCAT and VMAT plans in relation to their small aperture ratio (≤3.5 mm) are shown in Fig. 5. The 2%/2 mm and the 3%/3 mm passing rates are displayed in relation to the ratio of small apertures. A linear regression shows a general trend of deteriorating pass rates with increasing ratio of small apertures. Assuming linear correlation, the R^2^ values are 0.2229 and 0.436 for the 3%/3 mm and 2%/2 mm, respectively, as shown in Fig. 5. The Pearson correlation coefficient was determined to be −0.774 (*P*‐value 0.00189) and −0.835 (*P*‐value 0.00038) for the 3%/3 mm and 2%/2 mm data, respectively.

## 4 DISCUSSION

There are three major questions that should be asked when considering these data:

1. Why did the VersaHD^TM^ delivery system not generate an interlock when the described delivery error occurred?
2. Do such small gaps (3.5 mm or smaller) occur in clinical plans?
3. Does this impact pass rates for clinical plans?

The following discussion will consider these three questions and recommend further work that could be undertaken.

### 4.1 No interlock

The dynamic tolerance for leaf control is set to 1.0 mm in Integrity 4.0.4. The specifications for the Agility^TM^ claim a leaf position accuracy of 1mm at isocenter with a RMS of 0.5 mm.^14^ A minimum possible field size is not stated.

For the 1mm nominal gap the delivered width was up to 4.1 mm. The leaf positional error can exceed 1.5 mm for each leaf bank, which does not comply with Elekta’s stated tolerance of 1 mm. The control system should therefore generate an interlock for quadrupling the field size, as a systematic error in leaf positioning occurred during beam delivery. However, this expected interlock was not generated.

The Agility^TM^ leaf positions are calibrated to 6MV radiation field rather than light field edge. This allows delivery of smaller gap sizes off‐axis, and a consistent 4 mm gap size in close proximity to central axis. Because of this calibration procedure, the MLC control system needs to take into account the radiation field offset as a function of leaf position in order to avoid potential leaf collision. This is achieved by implementing a constraint curve for the isocentric leaf gap, which is called the static isocentric closed leaf gap constraint (SICLG). SICLG is a function of gap position, as described in Elekta’s document “Agility™ and Integrity™ R4.0.0 Information for Treatment Planning Systems.”^15^ This document describes the correct implementation of SICLG in the TPS. Based on the findings of this study, Elekta have released a field change order, advising customers of a potential SICLG violation when Monaco is used in step and shoot mode.^16^ However, as shown in this study, SICLG can also be violated by Monaco in dynamic mode, such as dMLC or VMAT delivery. The results of this study raise serious concerns about the implementation of the MLC control system. Obviously the MLC controller is successfully avoiding leaf collisions based on an implemented constraint curve, however, it does not provide any feedback to the user when leaf positions were changed during delivery in order to avoid leaf collisions. The ability of the MLC control system to adapt leaf positions without any warning can lead to violation of leaf position tolerances.

According to Rangel and Dunscombe systematic leaf positioning errors must be limited to 0.3mm in order to achieve delivered dose accuracy within 2 Gy for organs at risk and within 2% for the equivalent uniform dose to the target.^17^

As even EPID imaging verification tools can detect systematic MLC errors as small as 3 mm^18^ the expectation for the MLC controller would be to be far more sensitive to leaf positioning errors than this. However, the data presented in our study seem to suggest that the Agility^TM^ used in dynamic mode might not generate an interlock for errors as large as 1.5 mm.

Comprehensive analysis by Kerns et al. has determined a RMS leaf position accuracy of 0.32 mm for dynamic treatment fields.^19^ Wang et al. have found the leaf positioning accuracy on an Elekta Synergy linac largely to be within 0.5 mm.^20^ Both of these studies, however, have not focused on small apertures in dynamic mode.

### 4.2 Small gaps in clinical plans

Tatsumi et al. have analyzed the MU‐weighted segmental average of the mean leaf gap width and correlated this with average pass rates.^21^ Our study suggests that the minimum gap width, as prescribed by the TPS, may be a sensitive metric to predict pass rates. We have determined the MU‐weighted ratio of gaps that are 3.5 mm or less to the total amount of MU‐weighted apertures. For representative clinical VMAT plans we found this ratio to be anywhere between 0.05% and 10%. For more complex cases, like a single‐isocenter multiple brain metastasis treatment, this ratio can exceed 24% (see Fig. 5). Depending on the location of these 3.5mm gaps, they could introduce a relatively large error in delivered dose.

### 4.3 Impact on pass rates

Multiple studies have demonstrated the impact of MLC positioning errors on the accuracy of dose delivery.^22^, ^23^, ^24^, ^25^ Even though it was shown that VMAT is less susceptible to delivery errors than dMLC treatments,^26^ tight tolerance and action levels for modulated treatments are essential. In 2003 Palta et al. have suggested an action level of 0.5 mm gap width deviation.^27^ Oliver et al. reported on suitable action levels based on desired target coverage and recommended errors be kept within 0.6 mm.^28^ More recently, Rangel and Dunscombe proposed a 0.3 mm limit.^17^

This study demonstrates that the Agility^TM^ control system does not comply with these suggested limits. The same dynamic MLC sequence delivered on the TrueBeam linear accelerator shows good agreement. This indicates that the problem does not lie with the MLC parameters used by the TPS, but rather with the Agility^TM^ control system. This observation is also confirmed by a log file analysis.

The linear regression shown in Fig. 5 suggests that there could be some correlation between passing rates and ratio of small segments in any given plan. The relatively low R^2^ values of 0.2229 and 0.436 seem to indicate that there are most likely other factors involved causing such poor passing rates, which warrant further, more systematic investigations. A negative Pearson correlation coefficient also indicates deteriorating passing rates with increasing contribution of small segments.

The request by the TPS for small gaps in a patient treatment plan could lead to poor QA pass rates, especially when the MLC is not performing optimally, for example, with aging leaf motors or lack of adequate maintenance.

These errors might be large enough to have a dosimetric impact but not large enough to generate a machine interlock.

### 4.4 Future directions

There are multiple avenues that can be investigated. First, a multi‐institutional evaluation of planned vs actual leaf positions during delivery, as has been published already for Varian machines.^19^ Second, to introduce systematic and random MLC positional or speed errors in representative patient plans to determine the Agility^TM^ control system’s threshold for error detection. Subsequently, the impact on dose delivery and dose volume histogram parameters for representative patient plans can be quantified.

While all this work will certainly be interesting to undertake, we believe that the current state of this project warrants sharing among the wider Medical Physics community, as we have concerns about the performance of the Agility^TM^ control system.

Unless leaf positioning accuracy can be guaranteed during beam delivery, the possibility of mistreatments on the Agility^TM^ is very real, especially when a TPS does not take the identified Agility^TM^ limitations into account. Each center must therefore set appropriate action levels in their quality assurance program to identify potential delivery problems.

## 5 CONCLUSION

A deficiency of the Agility^TM^ control system was identified in this study, showing that beam delivery continues without interlock despite exceeding performance specifications. The resulting maximum dose difference at the central axis was observed to be up to 10.2% and 61.3% for a small field of 1 mm and 3.5 mm width, respectively. These findings are of concern and warrant imposing additional quality assurance measures to ensure patient treatment is as safe as possible.

## Conflict of Interest

No conflict of interest.
